# Expression of myogenic regulatory factors in chicken embryos during somite and limb development

**DOI:** 10.1111/joa.12340

**Published:** 2015-07-16

**Authors:** Gi Fay Mok, Rabeea Hazim Mohammed, Dylan Sweetman

**Affiliations:** ^1^ School of BiosciencesUniversity of NottinghamSutton BoningtonUK; ^2^ Present address: School of Biological SciencesUniversity of East AngliaNorwichUK

**Keywords:** chicken embryo, MRF, *MRF4*, *Myf5*, *MyoD*, myogenesis, *myogenin*

## Abstract

The expression of the myogenic regulatory factors (MRFs), *Myf5*,* MyoD, myogenin* (*Mgn*) and *MRF4* have been analysed during the development of chicken embryo somites and limbs. In somites, *Myf5* is expressed first in somites and paraxial mesoderm at HH stage 9 followed by *MyoD* at HH stage 12, and *Mgn* and *MRF4* at HH stage 14. In older somites, *Myf5* and *MyoD* are also expressed in the ventrally extending myotome prior to *Mgn* and *MRF4* expression. In limb muscles a similar temporal sequence is observed with *Myf5* expression detected first in forelimbs at HH stage 22, *MyoD* at HH stage 23, *Mgn* at HH stage 24 and *MRF4* at HH stage 30. This report describes the precise time of onset of expression of each MRF in somites and limbs during chicken embryo development, and provides a detailed comparative timeline of MRF expression in different embryonic muscle groups.

## Introduction

The myogenic regulatory factors (MRFs) are a group of four helix‐loop‐helix transcription factors that are critical regulators of muscle cell commitment and differentiation in vertebrates (Pownall et al. 2002; Chanoine et al. 2004; Berkes & Tapscott, 2005; Tapscott, 2005; Sweetman, 2012). The discovery of *MyoD*, based on its ability to convert fibroblasts into muscle cells (Davis et al. 1987), was soon followed by the identification of other closely related genes that share this transformative property: *Myf5, myogenin (Mgn)* and *MRF4* (Braun et al. 1989, 1990; Edmondson & Olson, 1989; Rhodes & Konieczny, 1989; Wright et al. 1989; Fujisawa‐Sehara et al. 1990; Miner & Wold, 1990). Together these genes orchestrate the formation of all skeletal muscle in the vertebrate embryo.

As these genes have such critical roles in the development of muscle, their regulation and expression has been extensively studied. A series of mouse reporter lines have been used to uncover a large number of complex interdigitated enhancers that regulate *Myf5* and *MRF4* expression, which are distributed across 150 kb around these closely linked loci (Tajbakhsh & Buckingham, 1995; Tajbakhsh et al. 1996; Summerbell et al. 2000; Carvajal et al. 2001, 2008; Buchberger et al. 2003, 2007; Teboul et al. 2003). In mammals *MyoD* expression is primarily controlled by two enhancers, the core enhancer and distal regulatory region, which regulate the onset and maintenance of *MyoD*, respectively (Goldhamer et al. 1992; Tapscott et al. 1992; Asakura et al. 1995; Faerman et al. 1995; Chen et al. 2001, 2002; Chen & Goldhamer, 2004), and similar regulatory sequences have been identified in birds (Pinney et al. 1995). *MyoD* in turn regulates expression of *Mgn* and can interact directly with its promoter to recruit chromatin remodelling complexes (de la Serna et al. 2005; Armand et al. 2008; Deato et al. 2008).

Understanding of development has been greatly informed by the use of the chicken embryo as a model organism (Tickle, 2004; Davey & Tickle, 2007), and it has been an exceptionally useful system to understand the developmental signals that regulate myogenesis in different embryonic domains, such as the epaxial myotome (Münsterberg et al. 1995; Borycki et al. 1998; Schmidt et al. 2000), the hypaxial myotome (Dietrich et al. 1998; Dietrich, 1999) and the developing limb (Dietrich et al. 1999; Delfini et al. 2000; Edom‐Vovard et al. 2001; Marics et al. 2002; Geetha‐Loganathan et al. 2005; Mok et al. 2014).

However, the existing literature does not comprehensively define the stages and expression patterns of each MRF during early somite and limb development in chicken embryos, although this has been done for the head musculature (Noden et al. 1999). To address this, the expression of each MRF has been compared in somites and limb using *in situ* hybridisation to delineate the relative timings and expression domains of each of these genes.

## Materials and methods

Dig‐labelled probes for *in situ* hybridisation were generated and used as described (Sweetman et al. 2008). Fertilised white leghorn eggs were obtained from Henry Stewart (Norfolk) and incubated to reach the desired stage.

Briefly, embryos were collected, staged (Hamburger & Hamilton, 1951), fixed in 4% paraformaldehyde (PFA) at 4 °C overnight then dehydrated by washing in 50% methanol/phosphate‐buffered saline (PBS) with 0.1% Tween (PBSTw) then twice with 100% methanol and stored at −20 °C. Embryos were rehydrated by washing in 75%, 50% and 25% methanol/PBSTw then washed twice in PBSTw. Embryos older than HH stage 20 were treated with proteinase K in PBST at 10 μg mL^−1^ for 25 min, then rinsed twice in PBST and fixed in 4% PFA/0.1% glutaraldehyde for 20 min at room temperature followed by two washes in PBSTw. Embryos were then washed in 1 : 1 PBSTw : hyb solution (50% formamide, 1.3 × SSC pH 5, 5 mm EDTA, 50 μg mL^−1^ yeast RNA, 0.2% Tween‐20, 0.5% CHAPS, 100 μg mL^−1^ heparin), washed with hyb solution for 10 min, then incubated in fresh hyb solution incubate at 65 °C for at least 2 h. Probes in pre‐warmed hyb solution at 65 °C were added and incubated overnight at 65 °C.

Embryos were rinsed twice in hyb solution at 65 °C, washed for 10 min in hyb buffer at 65 °C, then washed twice for 30 min in washing buffer (50% formamide, 1 × SSC pH 5, 0.1% Tween‐20) at 65 °C. Embryos were washed for 10 min at 65 °C in 1 : 1 washing buffer : MABT (100 mm maleic acid, 150 mm NaCl, 0.1% Tween‐20, pH 7.5), rinsed three times in MABT, and washed twice for 30 min in MABT. They were then blocked in 2% Roche blocking reagent (cat no. 11096176001) in MABT for 1 h, then in 2% Roche blocking reagent/20% goat serum in MABT for 4 h. Anti‐Dig‐AP Fab fragments (Roche, cat no. 11093274910) were diluted 1 : 2000 in 2% Roche blocking reagent/20% goat serum in MABT and incubated overnight at 4 °C.

Embryos were washed three times for 1 h in MABT and then twice for 10 min in NTMT (100 mm NaCl, 100 mm Tris pH 9.5, 50 mm MgCl_2_, 1% Tween‐20). Colour was developed with 9 μL NBT (4‐nitro blue tetrazolium chloride at 75 mg mL^−1^ in 70% dimethylformamide) and 7 μL BCIP (5‐bromo‐4‐chloro‐3‐indolyl‐phosphate, 4‐toluidine salt at 50 mg mL^−1^ in dimethylformamide) mL^−1^ of NTMT. After the staining reaction, embryos were de‐stained in high detergent mix, 5 × TBST (for 100 mL of a 5 × solution: 4 g NaCl, 12.5 mL 1 m Tris‐HCl pH 7.5, 0.1 g KCl, 5 mL Tween‐20) to reduce background and, if required, re‐stained.

For sectioning, embryos were dehydrated through 50%, 70%, 90% and 100% ethanol, washed in xylene them embedded in paraffin wax. Sections (10 μm) were cut, dewaxed in xylene and mounted in Depex for imaging.

## Results

### Expression in somites from HH stages 9 to 14

*Myf5* expression is first detected at HH stage 9 (6–8 somites) in the medial somite adjacent to the neural tube. Expression was observed in somites 1–6 in embryos with 7 and 8 somites, but Myf5 expression was not observed in embryos earlier than this. At HH stage 10, expression is seen in the anterior somites 1–6 (Fig. 1A), while the other MRFs are not expressed (Fig. 1B–D).

**Figure 1:**
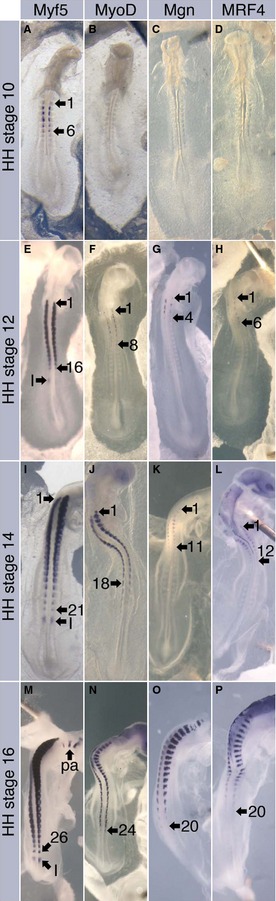
Expression of MRFs in early somite stage embryos. At HH stage 10, *Myf5* is expressed in somites 1–6 (A), while *MyoD* (B), *Mgn* (C) and *MRF4* (D) are not detected. At HH stage 12, *Myf5* is expressed in somites 1–10 (E), *MyoD* in somites 1–5 (F), but *Mgn* (G) and *MRF4* (H) are still not expressed. At HH stage 14, *Myf5* is expressed in somites 1–21 and in the presegmented mesoderm that will form the next somite (I). *MyoD* is expressed in somites 1–18 (J), *Mgn* in somites 1–10 (K) and *MRF4* in somites 1–8 (L). At HH stage 16, *Myf5* (M) is expressed in all somites and the presegmented mesoderm that will form the next somite, I, *MyoD* (N) is expressed in somites 1–24, *Mgn* in somites 1–20 (O) and *MRF4* in somites 1–20 (P).

At HH stage 12 (14–16 somites), *Myf5* is detected in all somites and, in some cases, paraxial mesoderm (Fig. 1E). *MyoD* was not detected in 14‐somite embryos but was in somites 1–8 in 2/5 15‐somite embryos and 16‐somite embryos (Fig. 1F), while *Mgn* and *MRF4* are expressed in anterior somites, respectively (Fig. 1G,H). The staining of *Mgn* and *MRF4* observed at these stages is variable. *Mgn* is detected in 5/10 16‐somite embryos: in two of these expression is seen in the two most anterior somites, in the other three of the four most anterior somites are stained. Similarly, in 16‐somite embryos *MRF4* is seen in 7/11 embryos: two have expression in somites 1–4, four in somites 1–6 and one in somites 1–8.

At HH stage 14 (20–22 somites), strong *Myf5* expression is seen in all somites and in somite position I, the region where a new somite is about to form. Posterior somites show *Myf5* restricted to the medial somites, while in more anterior somites expression is expanded through the whole medial–lateral extent. *MyoD* expression at this stage is seen in somites 1–18 and, like *Myf5*, is restricted to the medial region in more posterior somites but is more widespread in anterior somites (Fig. 1J), while *Mgn* and *MRF4* are now detected in somites 1–10/12 (Fig. 1K,L). Again there is some heterogeneity in the expression of *Mgn* and *MRF4* at these stages and, at a given somite number, differences in the staining pattern of up to two somites either more or less are observed.

At HH stage 16 (26–28 somites), *Myf5* is seen in all somites and in the paraxial mesoderm that is about to form a new somite, and *MyoD* is detected in all but the most recently formed somites (Fig. 1M,N), while *Mgn* (Fig. 1O) and *MRF4* are expressed in somites 1–20 (Fig. 1P). As is seen at earlier stages, there is a degree of variability in this staining and some embryos will have one additional somite expressing either *Mgn* or *MRF4*.

Notably, *Myf5* expression in the medial segmental plate mesoderm, where the next somite will form, position I, is not observed in all embryos examined at these stages (Fig. 1I,M). This was observed in 2/11 embryos at HH stage 8, 1/17 embryos at HH stage 9, 2/12 embryos at HH stage 10, 7/18 embryos at HH stage 11, 8/24 embryos at HH stage 12, and 6/11 embryos at HH stage 13 (see Fig. S1).

Transverse sections of HH stage 16 embryos show distinct localisation of MRF transcripts. In anterior somites *Myf5* is expressed in the dorso‐medial and ventero‐lateral lips of the dermomyotome, and throughout both the epaxial and hypaxial myotome that lies directly under the dermomyotome (Fig. 2A–C). In more posterior regions (i.e. somite 15), *Myf5* is only detected in the dorso‐medial lip of the dermomyotome and forming epaxial myotome (Fig. 2D). In the segmental plate *Myf5* expression is found in the medial unsegmented paraxial mesoderm immediately adjacent to the neural tube (Fig. 2E).

**Figure 2:**
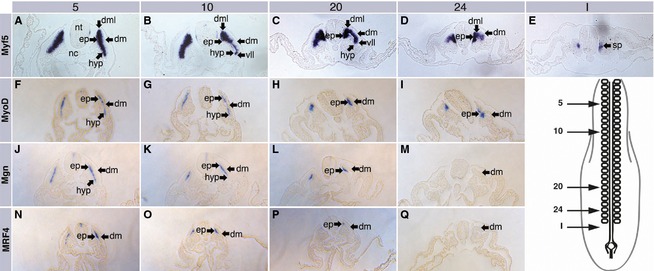
Transverse sections of HH stage 16 embryos stained with MRFs. In somites 5, 10 and 20, *Myf5* is expressed in the dorso‐medial lip of the dermomyotome as well as the whole of the myotome including both epaxial and hypaxial domains (A–C). In somite 24, expression of *Myf5* is only seen in the dorso‐medial lip and the epaxial myotome (D). In somite position I, *Myf5* is expressed in the medial presegmented mesoderm directly adjacent to the medial neural tube (E). *MyoD* is expressed in the epaxial and hypaxial in somites 5 and 10 myotome (F, G), and in more posterior somites this is restricted to the dorsal most region of the epaxial myotome (H, I). *Mgn* is expressed in both epaxial and hypaxial domains of anterior somites (J, K), and the epaxial myotome of somite 20 (L) but not in somite 24 (M), while *MRF4* (N–Q) is expressed in the epaxial myotome of somites 5, 10 and 20. dm, dermomyotome; dml, dorso‐medial lip of the dermomyotome; ep, epaxial myotome; hyp, hypaxial myotome; nc, notochord; nt, neural tube; sp, segmental plate; vll, ventro‐lateral lip of the dermomyotome.

*MyoD* in anterior somites is expressed throughout the myotome but not the dermomyotome (Fig. 2F–H), and in more posterior somites this is restricted to the dorso‐medial region of the myotome. In contrast to *Myf5*,* MyoD* is not detected in the dorso‐medial or ventro‐lateral lips of the dermomyotome.

*Mgn* is also expressed only in the myotome and not the dermomyotome, although it is restricted to the central domain in anterior somites and does not extend as far into the ventro‐lateral myotome as *MyoD* (Fig. 2J–M).

*MRF4* is also only expressed in anterior somites, but is only seen in the dorso‐medial part of the myotome (Fig. 2N–P) and does not extend into the ventro‐lateral myotome as far as either *MyoD* or *Mgn* at equivalent stages.

### Expression in somites from HH stage 20 to HH stage 26

Sections through older embryos (HH stage 20 to HH stage 26) at interlimb levels (i.e. between somites 22 and 25) show the myotome extending ventrally to form the trunk muscles. At HH stage 20, both *Myf5* and *MyoD* are expressed throughout the myotome (Fig. 3A,E), while *Mgn* and *MRF4* are expressed in a more restricted dorsal myotomal domain (Fig. 3I,M). At HH stage 22 and 24, *Myf5, MyoD* and *Mgn* are expressed in the ventrally extending myotome (Fig. 3B,C,F,G,J,K), while *MRF4* is still only expressed in the dorsal myotome. By HH stage 26, all four MRFs are expressed throughout the entire myotome (Fig. 3D,H,L,P).

**Figure 3:**
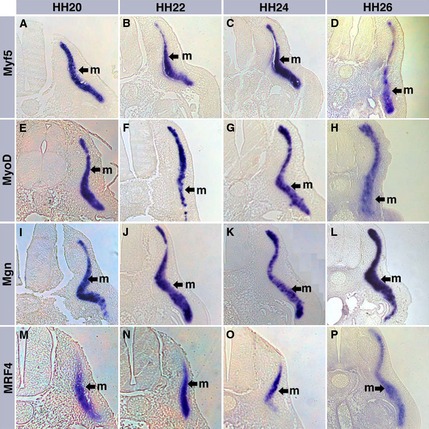
Transverse sections of somites at limb bud stages. *Myf5* is expressed through the whole myotome at HH stage 20–26 (A–D), as are *MyoD* (E–H) and *Mgn* (I–L). *MRF4* is expressed only in the central myotome at HH stage 20 to HH stage 24 (M–O), but expressed throughout the myotome at HH stage 26 (P). m, myotome.

### Expression in limb muscles

The muscles of the limb are derived from somitic migratory muscle precursors that express the transcription factors *Pax3* and *Lbx* (Dietrich, 1999; Otto et al. 2006). These cells delaminate from the ventro‐lateral lip of the dermomyotome then migrate into the developing limb where they form the dorsal and ventral muscle masses. Only at this point do they begin to express MRFs.

Whole‐mount *in situ* hybridisation shows *Myf5* expression in the forelimb at HH stage 22 (Fig. 4B), with expression in the hindlimb detected soon after at HH stage 24 (Fig. 4C). In sections, *Myf5* can be seen in both dorsal and ventral muscle masses in the forelimb at HH stage 22 (Fig. 5B), and weak expression can also be seen in hindlimb myogenic cells (Fig. 6B). *Myf5* expression is then maintained throughout the developing limb muscles at stages HH stage 24 to HH stage 30 (Figs 4D–F, 5C,D and 6C,D). At HH stage 30, *Myf5* staining becomes less intense, especially in distal muscles, as differentiation proceeds (Fig. 4f).

**Figure 4:**
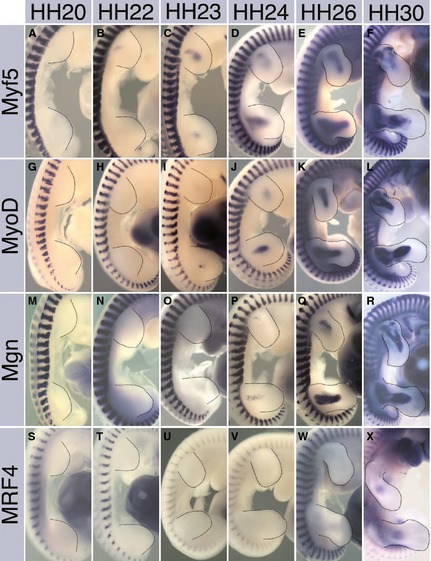
MRF expression in developing limbs. *Myf5* is first expressed in the forelimb at HH stage 22 (A, B), the hindlimb at HH stage 23 (C) and maintained until HH stage 30 (D–F). *MyoD* is first expressed in the hindlimb at HH stage 23 (G–I), then in both limbs to stage HH stage 30 (J–L). *Mgn* is first detected in both fore‐ and hindlimbs at HH stage 24 (M–P), and maintained through to HH stage 30 (Q, R), while *MRF4* is not detected in limb buds until HH stage 30 (S–X).

**Figure 5:**
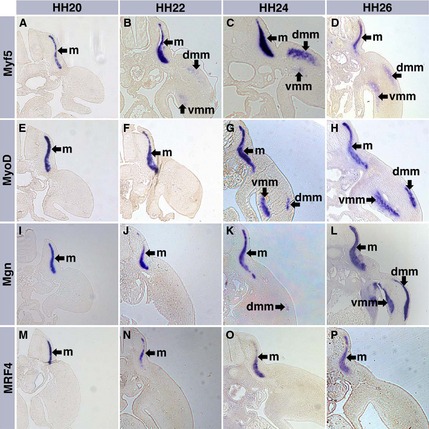
Transverse sections through forelimbs. *Myf5* is expressed in both dorsal and ventral muscle masses from HH stage 22 onwards (A–D), *Myod* from HH stage 24 (E–H), *Mgn* from HH stage 24 (I–L), while *MRF4* is not detected in limb buds at these stages (M–P). dmm, dorsal muscle mass; m, myotome; vmm, ventral muscle mass.

**Figure 6:**
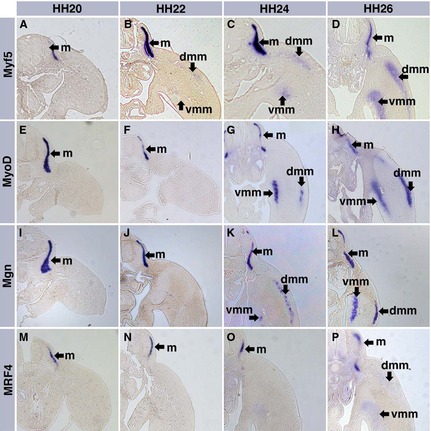
Transverse sections through hindlimbs. *Myf5* is expressed in both dorsal and ventral muscle masses from HH stage 22 onwards (A–D), *Myod* from HH stage 24 (E–H), *Mgn* from HH stage 24 (I–L). *MRF4* is not detected in limb buds at HH stages 20, 22 and 24 (M–O), but weak expression can be seen in both dorsal and ventral muscle masses at HH stage 26 (P). dmm, dorsal muscle mass; m, myotome; vmm, ventral muscle mass.

In contrast to *Myf5*,* MyoD* expression is first detected in dorsal and ventral muscle masses in the hindlimb at HH stage 23 (Fig. 4I), and then in the forelimb at HH stage 24 (Figs 3J, 5G and 6G). Expression is then maintained in the developing muscles throughout the limb up to HH stage 30 (Figs 4K,L, 5H and 6H).

*Mgn* is first seen in both fore‐ and hindlimbs at HH stage 24 (Figs 4P, 5K and 6K), and is then maintained to HH stage 30 (Figs 4Q,R, 5L and 6L). *MRF4* is not detected in limb muscles until muscle differentiation is well advanced at HH stage 30 (Fig. 4W), although some weak staining can be seen in sections through hindlimbs at HH stage 26 (Fig. 6P).

### Concluding remarks

The current data show a clear temporal progression in both somites and limbs where *Myf5* is expressed first followed by *MyoD*. Subsequently, *Mgn* and *MRF4* are expressed with similar dynamics. This is consistent with current models in which *Myf5* and *MyoD* are required for myogenic commitment while *Mgn* and *MRF4* regulate differentiation. Although *MRF4* expression has been reported earlier or contemporaneously with *Myf5* in mouse somites (Summerbell et al. 2002) and can act to specify muscles in the absence of both *Myf5* and *MyoD* (Kassar‐Duchossoy et al. 2004), the current data imply that this function is not conserved in chicken embryos where *MRF4* is only expressed later in myogenic development.

Expression of *Myf5* in the paraxial mesoderm in HH stages 8–14 in the −1 somite position was also observed. This is consistent with previous reports that have also observed *Myf5* expression prior to somite formation (Kiefer & Hauschka, 2001). However, it is apparent when comparing larger numbers of embryos at these stages that there is heterogeneity in these samples with some embryos showing this expression and others not. This is observed even in embryos harvested, processed and stained in a single batch. One possible explanation is that the *in situ* protocol produces variable results; however, staining in other tissues, such as the limbs, is far more reproducible. Therefore, we believe that a more likely explanation is that they are capturing embryos at subtly different stages and that those that do not show this expression have just formed the newest somite and *Myf5* expression is yet to be initiated in the paraxial mesoderm prior to somite formation. The regulation of *Myf5* during development is under the control of a large number of diverse enhancer elements reviewed in Francetic & Li (2011), which may help to explain this complex expression during somitogenesis.

A degree of heterogeneity in the staining of *Mgn* and *MRF4* in somites between HH stages 12 and 16 was also observed. This may simply reflect variable efficiency of staining in different embryos. However, this variability is still observed in embryos with the same somite number stained in a single batch, and is not apparent in limb buds where staining of all probes is highly consistent and reproducible. There are two possibilities for this: either the current protocol does not reliably detect weaker staining in more posterior somites in all cases or there is some variability in the precise timing of onset of these later MRFs between different embryos, possibly because these embryos do not come from an isogenic inbred line and the difference in time between stages is small; as new somites are added every 90 min the time between one HH stage and the next is only 4.5 h.

One notable difference between limb and somite staining is relative timing of *Mgn* and *MRF4* expression. In somites these genes are expressed at the same point, although the domain of *MRF4* within the myotome is more restricted than that of *Mgn*. In contrast, *Mgn* expression in limb muscles precedes that of *MRF4*. It is clear that different muscle groups have different regulatory networks driving their differentiation (Mok & Sweetman, 2011), and this may explain the distinct staining dynamics observed. Also clearly different shapes in myotomes at different axial levels are observed. At limb levels the myotome does not extend dorso‐medially to the same extent as it does in the interlimb region. This is presumably to facilitate the migration of limb muscle precursors, although limb level somites do also produce the pectoral muscles by myotomal extension (Beresford et al. 1978), so this difference in shape does not preclude this mode of myogenesis.

It is also apparent that the data presented do not entirely agree with some previous reports that have detected *Myf5* expression in primitive streak at HH stage 3 and paraxial mesoderm and HH stage 5 (Kiefer & Hauschka, 2001). Despite extensive staining, expression has not been seen in these early embryos. This is presumably due to differences in the staining protocols and probes used, and it may be that the current approach sacrifices some sensitivity for specificity.

One intriguing question that remains is to what extent the myoblasts in a particular region of the embryo are homogeneous or consist of distinct subpopulations with different characteristics. Previous reports have already identified differences in the expression domains of *Myf5* and *MyoD* within the dorsal and ventral muscle masses of the limb (Delfini et al. 2000), and ablation experiments have also suggested that there are *Myf5*‐independent (Gensch et al. 2008; Haldar et al. 2008) but not *MyoD*‐independent (Wood et al. 2013) myogenic lineages within the embryo, although this view has been challenged (Comai et al. 2014; Haldar et al. 2014). However, the current *in situ* hybridisations do not provide single cell resolution and distinct populations cannot reliably be identified, so the resolution of this question will require further work.

## Author contributions

DS designed the original study; DS, GFM and RHM contributed to experimental design; GFM and RHM performed experiments; and DS wrote the manuscript.
